# Primary Investigation of Phenotypic Plasticity in *Fritillaria cirrhosa* Based on Metabolome and Transcriptome Analyses

**DOI:** 10.3390/cells11233844

**Published:** 2022-11-30

**Authors:** Ye Wang, Huigan Xie, Tiechui Yang, Dan Gao, Xiwen Li

**Affiliations:** 1 Institute of Chinese Materia Medica, China Academy of Chinese Medical Sciences, Beijing 100700, China; 2 Nin Jiom Medicine Manufactory (Hong Kong) Limited, Hong Kong 999077, China

**Keywords:** phenomics, phenotyping, genes, *Fritillaria cirrhosa*, metabolome, transcriptome

## Abstract

Phenotypic plasticity refers to the adaptability of an organism to a heterogeneous environment. In this study, the differential gene expression and compositional changes in *Fritillaria cirrhosa* during phenotypic plasticity were evaluated using transcriptomic and metabolomic analyses. The annotation profiles of 1696 differentially expressed genes from the transcriptome between abnormal and normal phenotypes revealed that the main annotation pathways were related to the biosynthesis of amino acids, ABC transporters, and plant–pathogen interactions. According to the metabolome, the abnormal phenotype had 36 upregulated amino acids, including tryptophan, proline, and valine, which had a 3.77-fold higher relative content than the normal phenotype. However, saccharides and vitamins were found to be deficient in the abnormal phenotypes. The combination profiles demonstrated that phenotypic plasticity may be an effective strategy for overcoming potential stress via the accumulation of amino acids and regulation of the corresponding genes and transcription factors. In conclusion, a pathogen attack on *F. cirrhosa* may promote the synthesis of numerous amino acids and transport them into the bulbs through ABC transporters, which may further result in phenotypic variation. Our results provide new insights into the potential mechanism of phenotypic changes.

## 1. Introduction

Plant phenotypic plasticity is the ability of plants to acclimatize to a heterogeneous environment via the generation of alternative phenotypes while keeping the invariable genetic information [1,2]. Phenotypic variation can be reversible or last multiple generations through epigenetic silencing; however, organisms grow more slowly as their growth environment lacks sufficient nutrient supplements [3,4]. Evidence supports a positive association between climatic variability and plasticity in allocation, mainly between leaf morphology, size, physiology, and mean annual temperature [5]. These fitness phenotypes develop special morphological and functional mechanisms that persist under stressful or suitable conditions [1]. For example, the leaf trait plasticity of alpine plants is a vital mechanism for enabling better tolerance to changing climate by plants, with leaf area, leaf thickness, and specific leaf area displaying regular variation across an elevation gradient in the Sichuan Province of China [6]. Alpine species also show significant signs of local adaptation via the high phenotypic plasticity of key phenotypic traits, including morphotype, total fruit length, growth, and height, for persistence and survival at high altitudes with low temperature and humidity [7]. Undoubtedly, plant phenotypic plasticity has become a hot topic for predicting plastic responses to climate change [5,8,9]

*Fritillaria* species are perennial plants whose fresh or dried bulbs are effective natural medicinal herbs for the treatment of respiratory diseases; this is because these bulbs contain multiple bioactive components, such as alkaloids and terpenoids [10]. To date, the estimated production value of formulations containing the herb is USD 400 million per year. *Fritillaria cirrhosa* D. Don is an actively traded plant that is marketed globally alongside 17 other plants with medicinal properties [11,12]. However, owing to low reproduction rate and overexploitation, medicinal plants of the genus have been listed as Grade II State Key Protected Wild Plants in China. In addition, at least 4–5 years are required for the entire life cycle from vegetative growth to the reproductive stage, and seed propagation remains the main productive means for large-scale cultivation. Unfortunately, a mass of *F. cirrhosa* in the reproductive stage reverses to the vegetative stage by losing flowers and even aerial stem during long-period cultivation, thereby affecting the quality and yield, or leading to failing acquisition of the medicinal parts of *F. cirrhosa*. Thus, a systematic study is necessary to explain the potential mechanism underlying phenotypic variation in the growth process of *F. cirrhosa*.

To date, the phenotypic variation in *Fritillaria* plants has not been reported. The development of omics techniques has accelerated the genetic dissection of complex traits [13]. Metabolomic analysis simultaneously revealed vast metabolites that participate in the plant life process, of which the primary metabolites play an important role in regulating phenotypic variation and influencing the synthesis of secondary metabolites [14,15,16]. Transcriptome analysis can be used to interpret the molecular differences between plants with different phenotypes plants caused by plasticity. De Villemereuil et al. published a detailed pattern of phenotypic plasticity in the alpine plant, *Arabis alpina*, using two genome scanning approaches. These researchers found that sucrose phosphate synthase 1 is associated with altitude and local average temperature [7]. Accordingly, these two omics techniques can be utilized as an excellent approach for investigating the potential mechanism of phenotypic plasticity in *F. cirrhosa*.

In the present study, the variation in the normal and abnormal phenotypes of *F. cirrhosa* was evaluated in terms of metabolome and transcriptome data. The aims of this study were to (a) explore the leading metabolites that participate in the phenotypic plasticity of *F. cirrhosa*; (b) identify the main annotation pathways of differentially expressed genes that result in phenotypic variation in the plant; and (c) reveal the potential molecular mechanism by which long-year cultivation leads to *F. cirrhosa* from the vegetative growth stage to the reproductive stage via the integration of metabolome and transcriptome analyses. The data from this study not only provide a scientific theoretical basis for obtaining high-quality *F. cirrhosa* with a stable phenotype and large-scale cultivation but will also markedly benefit the improvement of future *F. cirrhosa* breeding. Herein, we hypothesized that the normal phenotype suffers from stress and leads to extremely small blubs, leading to failed germination of the aerial stem and the formation of an abnormal phenotype.

## 2. Materials and Methods

### 2.1. Preparation of the Plant Materials

Fresh bulbs and mature leaves of *Fritillaria cirrhosa* D. Don were collected from the Tu Autonomous County of Huzhu (36°59′ E, 101°59′ N, altitude: 3050 m), Haidong City, Qinghai Province, China. Three types of fresh bulbs were collected at the initial stage of germination of the aerial parts from 4- and 6-year-old plants whose bulbs from previous year still existed; these samples were labeled as four-year samples (FY), six-year samples (SY), and aberrant six-year samples (ASY). The bulbs were used to conduct the metabolome analysis to determine the primary metabolites of bulbs with different phenotypes. Three types of fresh leaves without any damage by insects or diseases were harvested at the seedling stage in which these plants no longer grew. These mature leaves were subjected to transcriptome analysis to identify vital differentially expressed genes (DEGs). All materials were stored at −80 °C until metabolic measurement and RNA extraction. Nine batches of blubs and leaf samples from the three growth phenotypes were used in the biological repetition experiments.

### 2.2. Metabolite Extraction and Metabolic Profiling

The *F. cirrhosa* bulbs were freeze-dried using a vacuum freeze-dryer (Scientz-100F, Scientz, Zhejiang, China) and crushed into a powder using a mixer mill (MM 400, Retsch, Haan, Germany) with zirconia bead for 1.5 min at 30 Hz. The obtained lyophilized powder (0.1 g) was extracted with 1.2 mL of 70% methanol (Merck, Darmstadt, Germany) solution for 30 s every 30 min (six times in total) under vortex shock. The extract was stored at 4 °C overnight and centrifuged at 12,000 rpm for 10 min. The supernatant was filtered using a 0.22 μm Millipore filter prior to injection into the ultra-performance liquid chromatography–electrospray ionization tandem mass spectrometry system (Agilent, New York, NY, USA). Standard substances (dimethyl sulfoxide) and formic acid of chromatographic purity were purchased from Merck (Darmstadt, Germany).

A Shimadzu Nexera X2^®^ LC system coupled with UPLC–ESI–MS/MS was used to analyze the metabolites in the extracts. Chromatographic separation was performed using an Agilent SB-C18 column (1.8 µm, 2.1 mm × 100 mm, Agilent, New York, NY, USA) at 40 °C. The mobile phase was composed of 0.1% (*v*/*v*) formic acid (Merck, Darmstadt, Germany) in water (A) and acetonitrile (B, Merck, Darmstadt, Germany) and the gradient was as follows: 5–95% B, 0–9 min; 95% B, 9–10 min; 5–95% B, 10–11 min; 5% B, 11–14 min. The flow rate was 0.35 mL·min^-1^ and the sample injection volume was 4 μL. The optimized ESI parameters were set as follows: source temperature, 550 °C; ion spray voltage, 5500 V (positive mode)/–4500 V (negative mode); and the collision-activated dissociation, high. The gas curtain, gas I, and gas II pressures were set to 25, 50, and 60 psi, respectively. The analyst software (Version 1.6.3, AB Sciex, Framingham, MA, USA) was used for the control +/– ion mode. Qualitative analysis was performed based on MS information recorded in multiple reaction monitoring modes. Spectral data were qualitatively analyzed using the Metware database (MWDB) of the Maiwei Metabolism Company (Wuhan, China) and the public database of metabolite information.

### 2.3. RNA Extraction and Library Construction

Total RNA was extracted from fresh mature leaves using RNAprep Pure Plant Hit (Beijing, China) according to the manufacturer’s protocol. The quality of the extracted RNA was determined via agarose gel electrophoresis. The RNA integrity and DNA mixture were determined using a Qubit 2.0 fluorimeter whereas a 2100 Bioanalyzer (Agilent Technologies, Santa Clara, CA, USA) was used to accurately determine the RNA concentration and integrity based on an RNA integrity number of 7.0. The isolation and enrichment of messenger RNA (mRNA) were completed using Oligo (dT). The mRNA was cut into short fragments after the addition of fragmentation buffer. cDNA was synthesized from the short fragments and purified using AMPure XP heads. After purification, polyA tails was added to both ends of cDNA and ligated with adapters, which are used for library construction before fragment selection and PCR enrichment. Library quality was analyzed based on accurate quality using the Q-PCR approach (effective concentration > 2 nM). Nine cDNA libraries were sequenced using Illumina HiSeq 2000 (NEB, San Diego, CA, USA) after pooling.

### 2.4. De Novo Transcriptome Assembly and Annotation of DEGs

Low-quality reads were removed based on three criteria using the FASTX Toolkit (Version 0.0.14, Hannon Lab, MD, USA): (1) reads containing adapters; (2) paired reads with N content exceeding 10% of the read base number; and (3) paired reads in which the number of low-quality (Q ≤ 20) bases in any sequencing read exceeded 50% of the number of read bases. High-quality reads were acquired after further detection of the sequencing error rate and GC content distribution were acquired, which were mapped into the assembled transcriptome to determine the expression quantity of genes due to the absence of reference genomes. After obtaining clean reads, the reference sequence was assembled using Trinity (version v2.6.6, Broad Institute of Massachusetts Institute of Technology and Harvard, Cambridge, MA, USA) [17].

Gene expression levels were calculated using the kilobase of transcript per million fragments mapped (FPKM) after normalizing the amounts of mapped reads and length of the transcript. The DESeq2 (Version 1.22.2) software was used to obtain a gene database of differential expression between different samples using reads without normalization [18,19,20]. DEGs were selected as |log2Fold change| ≥ 1 and false discovery rate (FDR) < 0.05. Functional analysis of DEGs was performed using public databases, including the Kyoto Encyclopedia of Genes and Genomes (KEGG), Gene Ontology (GO), and NCBI non-redundant protein sequences (Nr). The DEG sequences were compared with sequences in the public databases using BLAST software. The sequences of DEGs were aligned in the PinTFDB and PlantTFDB databases to identify the transcription factor (TF) families and transcriptional regulators (TFs).

### 2.5. Data Analysis and Statistics

Correlation analysis is a critical step in the assessment of the association between biological replicates to avoid the effects caused by biological variability. Herein, Pearson’s correlation coefficient was used as the assessment index. Chemometric analysis, such as principal component analysis (PCA) and orthogonal partial least squares discriminant analysis (OPLS-DA), was performed using the R package. PCA is classical chemometrics for non-parametric cluster analysis of cluster patterns and sample classification. PCA uses several PC linear to the original dataset and contains most of the original information. OPLS-DA is a supervised pattern recognition method that is combined with orthogonal signal correction and PLS-DA, often for significantly different metabolites and unigenes, and is used for the select different metabolites/genes based on variable importance in projection (VIP) ≥ 1 [21].

**Figure 1:**
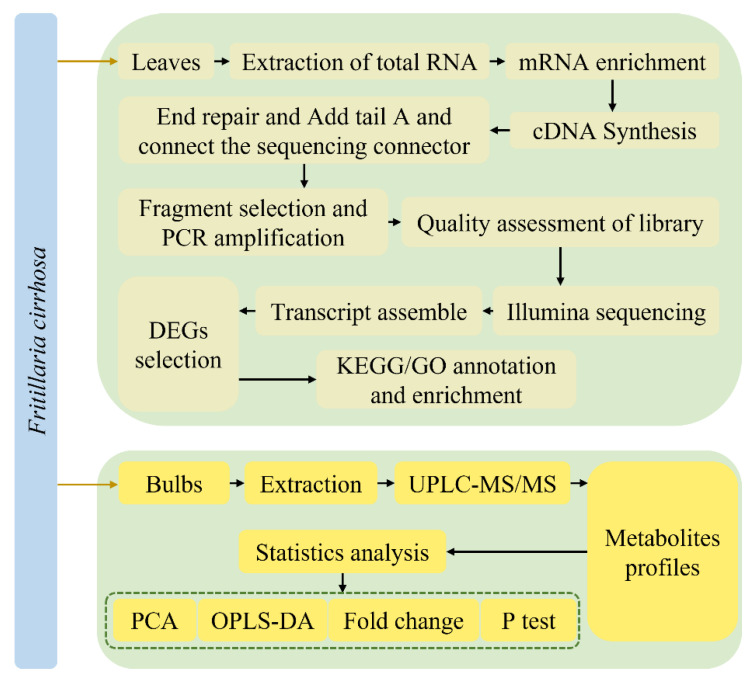
The experimental flow chart.

The metabolome and transcriptome analyses were performed in triplicate illustrated in Figure 1.

## 3. Results and Discussion

### 3.1. Morphological Characteristics of the Whole Life History and Phenotype Plasticity of F. cirrhosa

The whole life history of *F. cirrhosa* involves a long growth period from seeds to reproductive status [22]. The seeds of *F. cirrhosa* after post-ripeness germinate into weak leaves similar to pine needles in the first year, called Yi Ke Zhen (YKZ) in Chinese. The widish leaves, such as chicken tongue, can be obtained in the second year from first-year growth bulbs, called Ji She Tou (JST) in Chinese. Three-year-old *F. cirrhosa* are designated Yi Pi Ye (YPY) and they possess wider and longer leaves than two-year-old seedlings. Hereafter, the bulbs begin to germinate aerial stems with several leaves in the fourth year. Some individuals can flower but fail to produce fruit; the phenotype during this period is called Shu Er Zi (SEZ). Some four-year plants maintain the shape of three-year-old status. The five-year bulbs become flowering individuals (Deng Long Hua, DLH) that can produce capsules containing vast seeds, which is the last step in the whole life cycle. The first four years represent the vegetative stage and involve the accumulation of a large number of nutrient substances with the swelling of the bulbs in preparation for the regenerative status of the fifth year. Many uncertainties may lead to the undesirable growth of the aerial part, and the biomass of bulbs will decrease even if insufficient for flowering. Similar published research revealed that altitude difference led to an allometric relationship between the bulb and leaf in *F. przewalskii* owing to variation in leaf and bulb traits, biomass, and biomass allocation [23].

**Figure 2:**
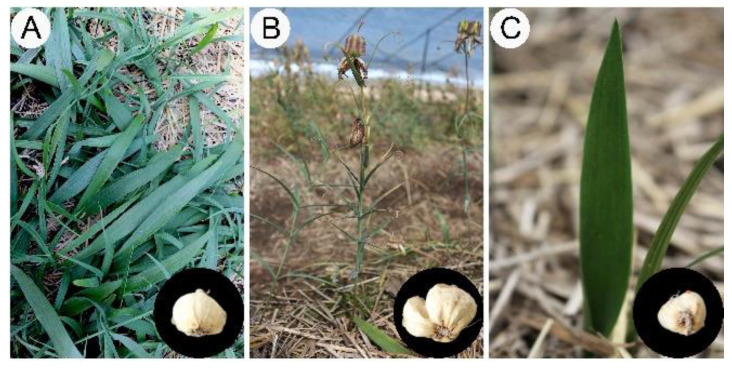
The morphological characters of the aerial parts and dried bulbs of *Fritillaria cirrhosa* at different growth years. (**A**) Four-year stages (FY); (**B**) six-year stages (SY); (**C**) six-year stages appeared phenotype variation (ASY).

Herein, we revealed an interesting phenomenon in which *F. cirrhosa* can produce morphological variation from DLH (Figure 2B) to a phenotype (Figure 2C) similar to YPY (Figure 2A) under potential stress conditions. The aerial botanical part with traits of sexual maturity reverted to the propagation stage, displaying a leaf shape similar to bamboo leaf; this is a typical characteristic of leaf shape in the three- or four-year stage (Figure 2). Furthermore, the bulbs exhibiting phenotypic plasticity were obviously decreased in terms of shape and biomass compared to bulbs in the same growth year, and the returned bulbs were similar to those of four-year plants. Undoubtedly, phenotypic plasticity would lead to the yield reduction of medicinal blub and reproductive seeds in the cultivation of *F. cirrhosa*. Therefore, we hypothesized that variations in cultivation conditions lead to changes in the accumulation of primary metabolites in bulbs and further changes in the morphological variation in the aerial parts. Accordingly, the normal phenotype suffers from potential stress and leads to extremely small blubs, resulting in failed germination of the aerial stem and the formation of an abnormal phenotype.

### 3.2. Metabolomic Results

#### 3.2.1. Variation in Primary Metabolites may Contribute to the Phenotype Plasticity of *F. cirrhosa*

**Figure 3:**
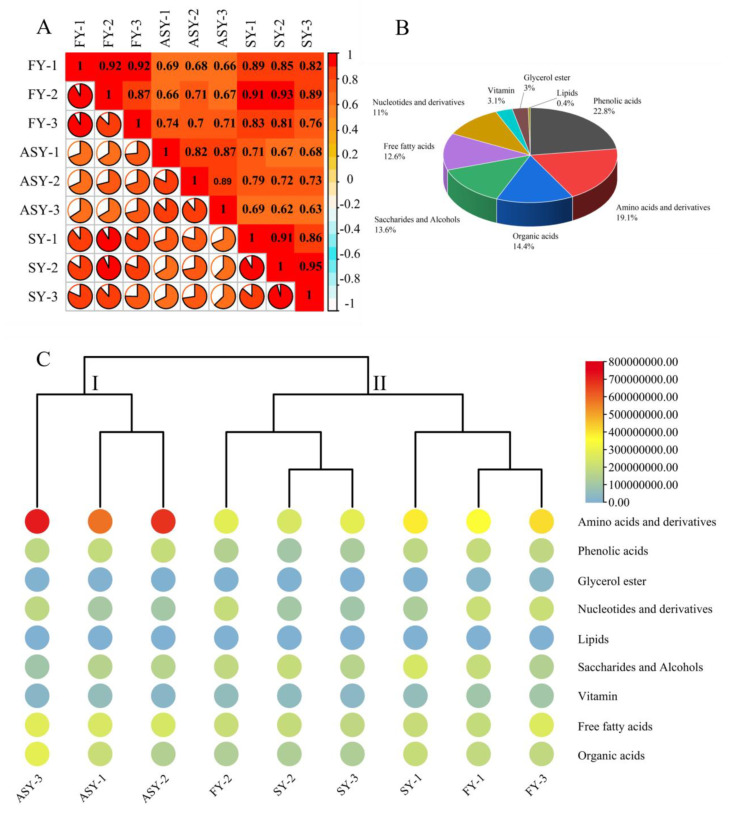
The metabolomics data reveal the change in primary metabolites among three phenotypes of *F. cirrhosa*. (**A**) Correlation analysis; (**B**) category of metabolites; (**C**) heatmap of metabolites. FY: four-year samples. SY: six-year samples. ASY: aberrant six-year samples.

Several metabolites participate in normal plant life processes, and metabolic regulation also is an important strategy under stress conditions during growth and development [24,25]. Maize was found to display a plastic response to heat or cold stress in terms of biomass allocation and metabolic plasticity, suggesting that shikimate and its aromatic amino acid derivative and non-polar metabolites play the main roles in adaption to temperature variations [26]. Sea buckthorn berries were found to display plasticity in their composition. In particular, ripe berries from Northern Finland contained higher levels of quinic acid and ascorbic acid than those from Southern Finland and Canada [27]. Primary metabolites are the basal origins of secondary components, and a variation in the former has not been reported till now, especially in the phenotypes from the old stage (flowering stage) to the young stage (one-leaf vegetable status). Therefore, the extracts of freeze-dried powders were subjected to LC-MS/MS analysis to compare the variation in metabolite composition among the ASY, SY, and FY bulb samples. Before selecting the significantly altered metabolites between different growth periods, Pearson’s correlation coefficient (r) was calculated and employed as the assessment index of biological repetition; this coefficient can reflect the degree of difference between the groups. The r-value within the same group was higher than that outside the group, except for SY-2 whose value (0.91) was lower than that between SY-2 and FY-2 (Figure 3A), indicating that the metabolites obtained from the three comparable groups were reliable. In general, 508 metabolites were extracted and identified based on UPLC-MS/MS analysis and a self-building database (Table S1). These metabolites belonged to nine categories, of which phenolic acids (22.8%), amino acids and derivatives (19.1%), organic acids (14.4%), saccharides and alcohols (13.6%), free fatty acids (12.6%), and nucleotides and derivatives (11%) had high percentages of these compounds (Figure 3B).

A heatmap combined with a tree cluster was used to compare the chemical variations among the three *F. cirrhosa* phenotypes. The total relative contents of amino acids and their derivatives were higher than those of the other eight classes of components. Furthermore, the content of the amino acids (and their derivatives) in ASY was higher than that in the other two phenotypes (Figure 3C). No significant differences were found among the three phenotypes of *F. cirrhosa* in terms of glycerol esters, organic acids, phenolic acids, saccharides, and alcohols. The contents of amino acids and derivatives, and free fatty acids in ASY bulbs were significantly higher than those in the other two phenotypes. However, the total content of nucleotides and derivatives of ASY was obviously lower than that of FY, whereas no significant difference was observed for SY, which had the same cultivation stages. The relative vitamin content in ASY displayed a similar tendency to that of nucleotides and derivatives. The same comparison results indicated that the FY phenotype has short growth and strong vitality. The *F. cirrhosa* samples could be divided into two clades based on these constituents, in which ASY was solely classified into a clade. The cluster performance indicated that phenotype plasticity contributed to the two clades, which had a greater impact on the primary metabolites than the difference in growing years. Nonetheless, explaining the difference more precisely between individual *F. cirrhosa* in the same cluster was found to be a complication of HCA. Thus, PCA and OPLS-DA should be considered for further evaluation of sample clustering based on different phenotypes.

#### 3.2.2. Individual Cluster Performance of ASY Compared with That of SY and FY

**Figure 4:**
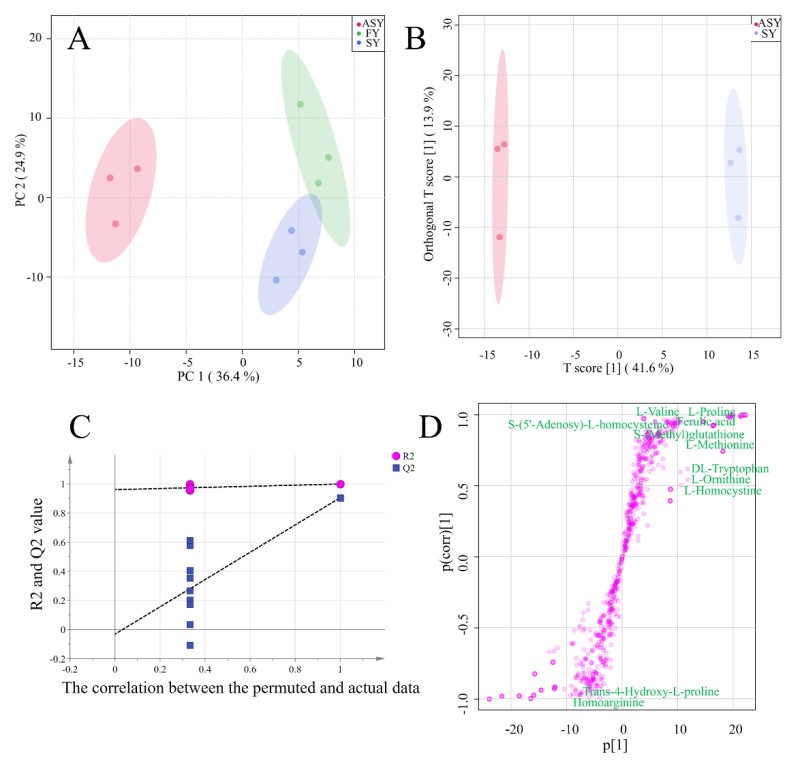
The results of unsupervised recognition and supervised recognition of metabolic data of *F. cirrhosa*. (**A**) Principal component analysis among three phenotypes; (**B**) score plot of OPLS-DA between ASY and SY; (**C**) the results of 200 times permutation test of OPLS-DA; (**D**) S-plot of OPLS-DA. FY: four-year samples, SY: six-year samples, ASY: aberrant six-year samples.

For a visual comparison of the three phenotypes of *F. cirrhosa*, a score plot was used for cluster analysis to compare the metabolite differences among the three phenotypes. The first two components explained 61.3% of the chemical variables. Based on PC1, 36.4% of the information could distinguish ASY from FY and SY samples (Figure 4A). The two-dimensional plot indicated obvious differences in the metabolites from the bulbs of *F. cirrhosa* after phenotypic plasticity. However, the classification failed to distinguish between the FY and SY samples owing to the similar class or content of chemical components. The failed cluster performance was consistent with the HCA and correlation analysis results (Figure 4A), which revealed a high r-value between the FY and SY samples. These results demonstrated that the difference in metabolite accumulation caused by phenotypic variation exceeds the change caused by years of growth.

In addition to the unsupervised recognition method, a supervised recognition approach was further developed to discriminate among the three phenotypes owing to the inferior cluster performance of PCA, in which the clusters between FY and SY were unclear. The score plot of OPLS-DA revealed that ASY and SY were clustered into two groups, and the 200 times permutation test indicated that the model was excellent as the high Q^2^ (0.91) and intercept of Q^2^ (−0.03) was below zero (Figure 4B,C). The S-plot indicated the important variables that contributed to the classification of the different clusters in OPLS-DA (Figure 4D). Eleven amino acids and their derivatives were found to be distributed at both ends of the S-plot, including l-methionine, l-ornithine, l-homocystine, dl-tryptophan, l-proline, l-valine, etc. In addition, l-methionine was among the top 20 most important variables. The cluster performance of OPLS-DA between ASY and FY is displayed in Figure S1. Notably, the robust model could distinguish the two phenotypes (Figure S1A). According to the S-plot (Figure S1B), the top 50 metabolites with significant differences included 8 amino acids and their derivatives which were obtained from a robust model with high Q^2^ (Figure S1C).

#### 3.2.3. Main Components of Amino Acids Contribute to ASY Phenotype Variation

**Figure 5:**
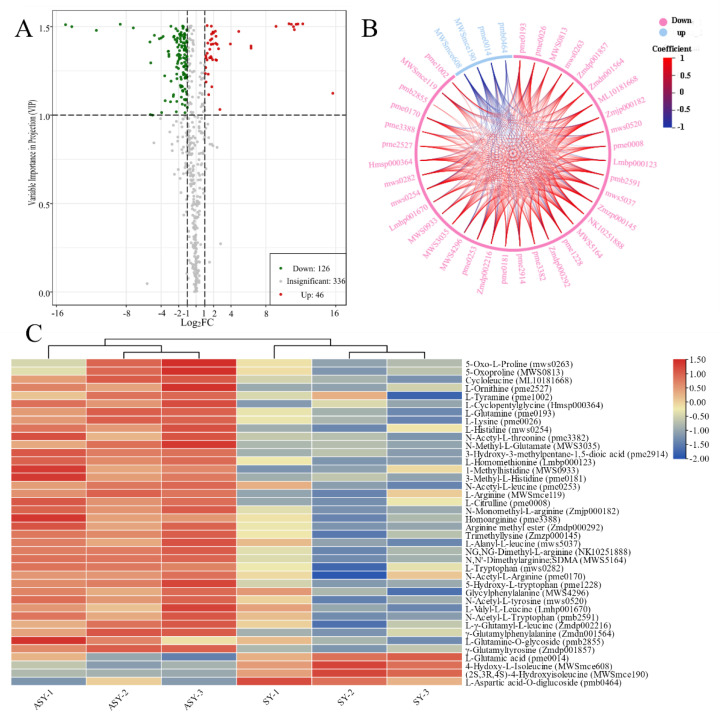
The selection of different metabolites and comparison of amino acids and derivatives between ASY and SY phenotypes of *F. cirrhosa*. (**A**) The volcano plot; (**B**) chord diagram of differential amino acids metabolites, which full names are the same as those of heatmap; (**C**) heatmap of amino acids metabolites. ASY: aberrant six-year samples. SY: six-year samples.

Volcano plots were used to display the different metabolites based on statistics and included all significant, upregulated, and downregulated components. In addition to the total relative content, the significant differences in metabolites with upregulated and downregulated profiles were used to select the potential metabolite marker category to reveal the potential mechanism for the phenotypic plasticity of *F. cirrhosa* (Table 1). The detection of different metabolites was conducted based on fold-change (2 ≤ value ≤ 0.5) and VIP value (≥1). Detailed information on the primary metabolites between ASY and SY is provided in Table S2, and a volcano plot based on the differences in metabolites between ASY and SY is presented in Figure 5A. A total of 172 significantly different metabolites were obtained between ASY and SY, of which ASY has 126 were upregulated and 46 were downregulated. Based on the results, the upregulated amino acids and derivatives may be the main reason for the occurrence of phenotypic plasticity in *F. cirrhosa* from the normal flowering stage to the one-leaf phenotype. A total of 36 amino acids and their derivatives were upregulated in ASY. These upregulated and downregulated amino acids and their derivatives had synergetic and antagonistic correlations in the bulb growth of *F. cirrhosa* (Figure 5B). An HCA cluster heatmap based on the relative content of amino acids and derivatives was constructed between ASY and SY, which revealed that most of the types of compounds had higher content than SY, except for four constituents: L-glutamic acid, 4-hydroxy-L-isoleucine, (2S,3R,4S)-4-hydroxyisoleucine, L-aspartic acid-O-diglucoside (Figure 5C). Among these, L-glutamic acid was reported to be a potential component beneficial to the yield of the hypogeal part [28] and stress tolerance [29], aligning with the field growth data that SY has higher bulb biomass and resistance than ASY.

A total of 166 significantly different components were found between ASY and FY, in which ASY had 88 downregulated metabolites and 78 upregulated chemical components (Figure S2A). The upregulated and downregulated amino acids and derivatives also had synergetic and antagonistic correlations (Figure S2B), and the relative content of the type of components was higher in ASY than in FY (Figure S2C).

#### 3.2.4. KEGG Annotation of the Significantly Different Metabolites Validates the Role of Amino Acids in the Phenotype Plasticity of *F. cirrhosa*

**Figure 6:**
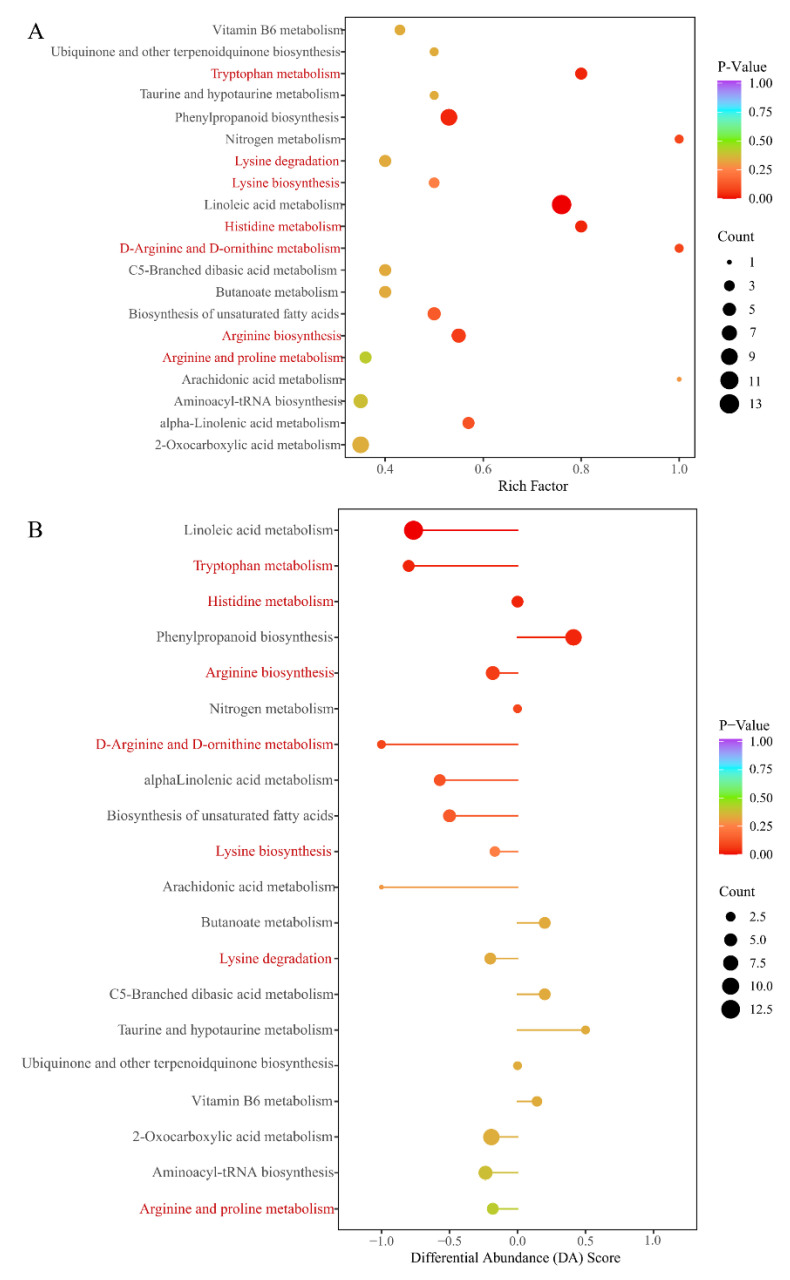
The main annotation results of different metabolites between ASY and SY phenotypes of *F. cirrhosa*. (**A**) The KEGG enrichment analysis; (**B**) differential abundance score. ASY: aberrant six-year samples. SY: six-year samples.

KEGG annotation of the significantly different metabolites between ASY and SY of *F. cirrhosa* is shown in Figure S3. These metabolites mainly participate in metabolism, genetic information processing, and environmental information processing. According to the classification results, most of the significantly different metabolites had a powerful relationship with metabolic pathways and biosynthesis of secondary metabolites in the comparison groups. The KEGG annotation results revealed that 15.79% of the metabolites were concentrated on the biosynthesis of amino acids, aligning with the content comparison mentioned above (Figure 6A). Enrichment analysis was performed to determine the percentage of metabolites with significant differences in total components using annotation information in the KEGG database. Herein, a high rich factor indicates a strong enrichment degree, and a low *p*-value reflects the enrichment significance. The top 20 pathways with the lowest *p*-value are shown in Figure 6A. There were seven pathways related to the biosynthesis and metabolism of amino acids and derivatives, including four metabolic pathways (tryptophan, histidine, D-arginine/D-ornithine, arginine, and proline metabolism), two biosynthesis pathways (lysine and arginine biosynthesis), and the lysine degradation pathway. Differential abundance score is an effective analytical index for assessing the variation of metabolites in the specific pathways; a positive value indicates upregulated expression while negative values reflect downregulated expression. Further, the line in the x-axis represents the expression positive (Figure 6B). The metabolic pathways of tryptophan, linoleic acid, nitrogen, histidine, and D-arginine/D-ornithine were identified as the main pathways between ASY and SY. The expression of metabolites pathways annotated in the tryptophan, linoleic acid, and D-arginine/D-ornithine metabolism pathways was downregulated. However, a clear tendency was not found for the other two pathways. The biosynthesis pathways of amino acids indicated the enrichment of the pathway mainly regulated by some upregulated (2-oxoglutarate and glutamate) and downregulated amino acids (lysine, arginine, lysine, citrulline, histidine, and five other components) (Figure S4).

The classification plot of KEGG annotation based on the differences in metabolites between ASY and FY is shown in Figure S5. The most obvious difference was a sulfur relay system in genetic information processing and plant hormone signal transduction in the annotation compared with that of the comparison groups of ASY and SY. This variation could be interpreted as ASY and FY having similar leaf shapes but different growth ages. Furthermore, 21.33% of the metabolites were annotated in the biosynthesis of amino acids. The percentage difference between ASY and SY and ASY and FY was caused by the number of amino acids and derivatives in the two groups; there were 40 amino acids and derivatives between ASY and SY, and 50 components of the same types between ASY and FY. KEGG enrichment analysis results and differential abundance score based on the different metabolites are displayed in Figure S6A and S6B, respectively. Herein, the biosynthesis of amino acids remained as one of the main enrichment pathways, with low *p*-values and high counts. The other enriched pathways related to amino acids were the same as those in ASY and SY. The differential abundance score indicated that most pathways had a score of >1.

Primary metabolism is tightly regulated by multiple environmental factors, and light and nutrient availability can lead to fluctuations in relative metabolites [30]. The interaction between primary metabolites and circadian rhythms enabled organisms to predict seasonal changes [30], validating nitrogen metabolism as the only differential KEGG pathway after enrichment analysis between ASY and SY. Amino acids have been suggested to play an essential role in plant defense response during biotic or abiotic stress [31]. Herein, the DL-tryptophan, L-proline, and L-valine results were consistent with the conclusion that plants sustain biotic stress. The amino acids and derivatives, and free fatty acids had significantly higher contents in the ASY bulbs than in the other two phenotypes. The accumulation of sucrose is often associated with stress response, which is linked to macromolecule stabilization and the membrane [32]. The present comparative research revealed that seven components of saccharides and alcohols and one nucleotide derivative (5’-diphospho-D-glucose uridine) were annotated in the pathways of starch and sucrose metabolism in the comparison groups of ASY and SY and ASY and FY. The annotation results confirmed our previous hypothesis that the phenotypic plasticity of *F. cirrhosa* is a survival strategy that adapts to stressful environments. Nevertheless, no sulfur relay system and plant hormone signal transduction information were found in the KEGG annotation category between ASY and SY, this could be interpreted as a low correlation between the phenotypic variation from the flowering status to the one-leaf stage and the two metabolism pathways. In addition, the main difference between FY and SY was the relative content of amino acids and derivatives, nucleotides and derivatives, and vitamins, which is a common phenomenon exhibited by bulb samples of *Allium*, where metabolites display significant cultivation year-specific differences [33].

### 3.3. Transcriptome Results

#### 3.3.1. Quality Assessment of Sequence Data and DEGs Comparison among the Three Phenotypes of *F. cirrhosa*

Based on quality assessment of the RNA extraction, the extracted concentrations were between 68 and 201 ng·μL^−1^, with integrity from 7.0–7.9, indicating excellent RNA quality and that the RNA met the standards for sequencing and library construction (Table S4). A total of 6.78 Gb, 6.89 Gb, and 6.65 Gb of sequence data were obtained from the fresh leaves of the three phenotypes. A total of 44,660,000–49,932,362 bp raw reads were obtained, and the length of the clean reads was between 42,725,158 and 47,591,740 bp. Q20 exceeded 97.61% while Q30 exceeded 93.46% with a 0.03% error rate. The GC content of all samples was approximately 50% (Table S5).

**Figure 7:**
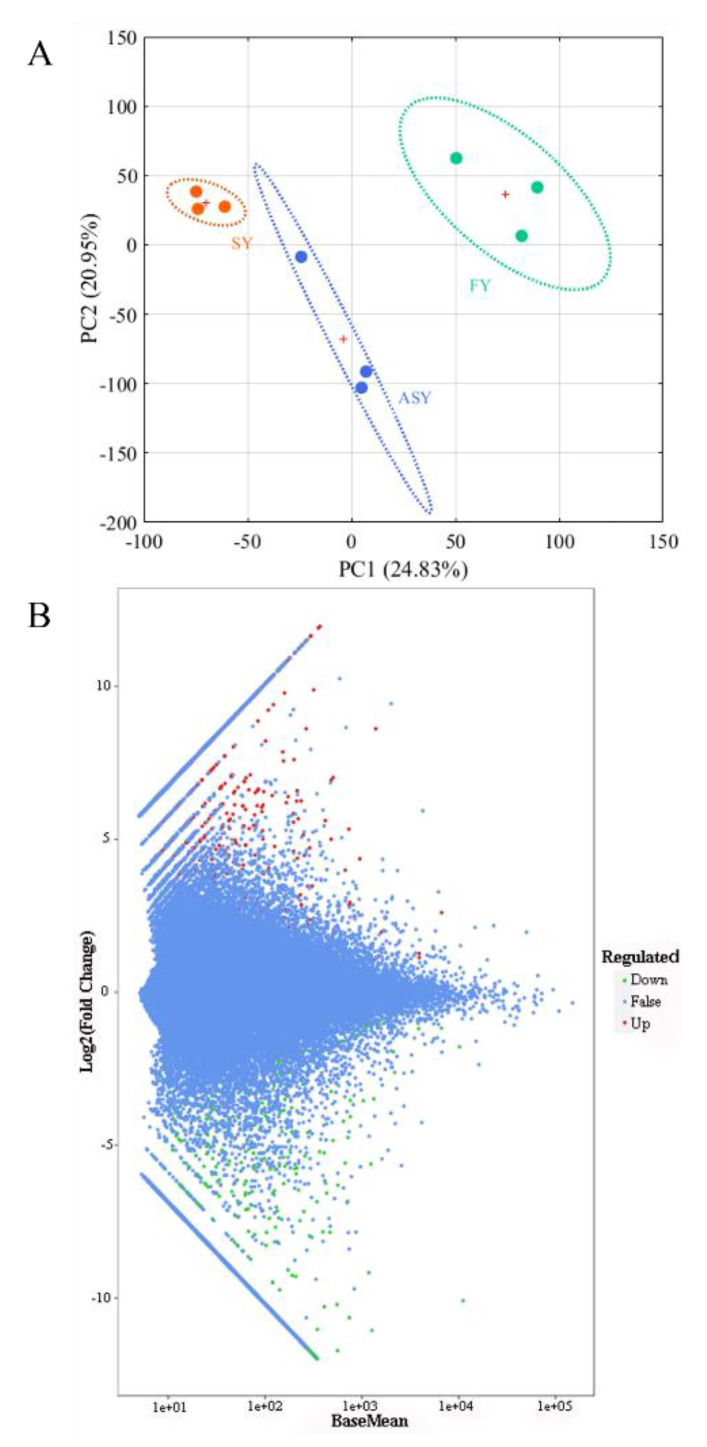
The unsupervised recognition and selection of different genes using sequencing data of *F. cirrhosa*. (**A**) The score plot of PCA; (**B**) the MA plot. SY: six-year samples. FY: four-year samples. ASY: aberrant six-year samples.

As the genome data of the *Fritillaria* genus were too large for sequencing [34], assembly of a large genome was extremely difficult [35,36]. Therefore, Illumina-based sequencing was conducted to analyze the *F. cirrhosa* transcriptome. A transcriptomic comparison was carried out to identify the DEGs among the ASY, SY, and FY samples to explain the potential mechanism resulting in the phenotypic plasticity of *F. cirrhosa*. The selection of different genes was conducted with DESeq2 using the count data. The selection criterion was |log2Fold change| ≥ 1 and FDR < 0.05, after correction by multiple hypothesis testing of Benjamini–Hochberg to the probability of hypothesis testing (*p*-value). A total of 17,494 DEGs were obtained in the three groups. PCA was carried out according to the FPKM values of these DEGs, and the two-dimensional score plot revealed a clear cluster tendency of the three phenotypes (Figure 7A). Herein, PC1 explained 24.83% of the variable information, separating SY leaves from FY leaves, and PC2 explained 20.95% of the variable differences, distinguishing ASY from the other two groups. The classification of ASY between SY and FY was consistent with the fact that the growth status of ASY matched the degenerated stage of SY; however, ASY had longer cultivation years than FY. DESeq2 was also applied to statistically analyze the number of total difference genes, up-, and downregulated genes in the two comparison groups: ASY and SY and ASY and FY using an M-versus-A (MA) plot, which served as a visualization method of data distribution using M (log2 (fold change)) and mean/average (A) (Figure 7B). There were 1696 DEGs between ASY and SY, of which 994 genes were upregulated and 702 genes were downregulated (Table S6). A total of 2137 DEGs were obtained from the comparison groups of ASY and FY, which comprised 1117 upregulated and 1020 downregulated genes (Table S7). In general, there were 1,366,332,329 special DEGs belonging to ASY and SY, and ASY and FY, respectively, whereas 76,323 DEGs belonged to ASY, FY, and SY, respectively (Figure S7).

**Figure 8:**
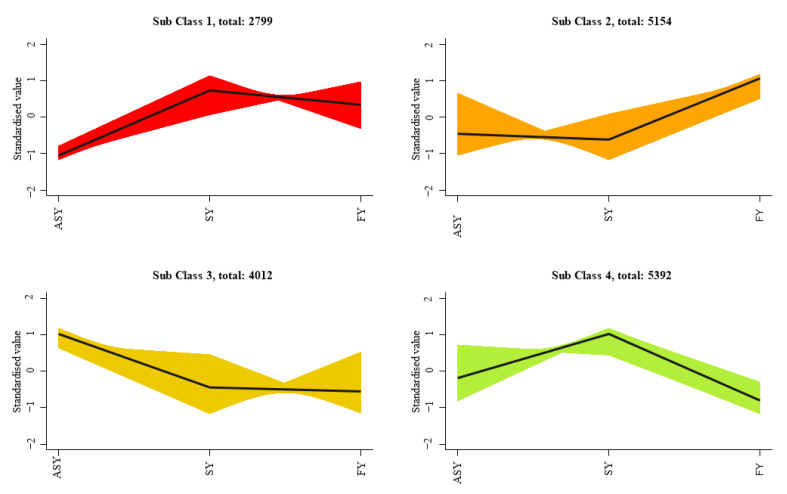
The K-mean classification analysis of gene expression in *F. cirrhosa*. SY: six-year samples. ASY: aberrant six-year samples. FY: four-year samples.

To better display the expression differences of similar genes among the three phenotypes of *F. cirrhosa*, K-mean classification was employed to compare the expression differences among the four classes of ASY, SY, and FY. The results indicated noticeable fluctuations in the differential expression (Figure 8). Sub-classes 1 and 4 comprised 2799 and 5392 genes, respectively, with SY having the highest gene expression relative to the other two phenotypes. ASY had the highest expression in sub-class 3, including 4012 genes, whereas the FY phenotype of *F. cirrhosa* had 4154 highly expressed genes in sub-class 2.

#### 3.3.2. Annotation Results of DEGs Show the Potential Reasons for the Phenotype Plasticity of *F. cirrhosa*, including the Biosynthesis of Amino Acids

A total of 392,652 unigenes were annotated in different databases (Nr, KEGG, and GO) containing at least 27% of gene information. In general, 178,254 unigenes, accounting for 45.40% of the total unigenes, were annotated in at least one database. According to the annotation results of the Nr database (Table S8), 536 unigenes were annotated into 13 *Fritillaria* species, of which 201 genes were consistent with those of *F. cirrhosa*, most of which displayed an associated with cytochrome P450, hydroxymethylglutaryl-CoA synthase, and some information in the chloroplasts of the species.

**Figure 9:**
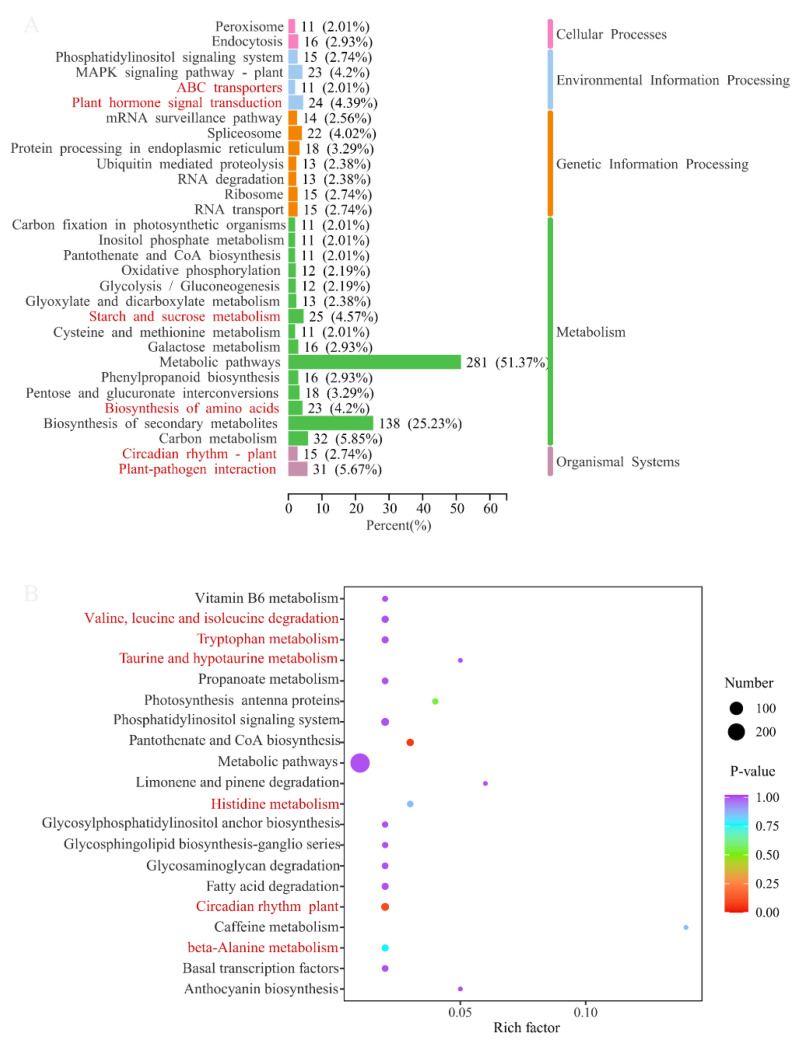
The main annotation results of difference genes between ASY and SY in *F. cirrhosa*. (**above**) KEGG classification analysis. (**below**) The enrichment analysis. ASY: aberrant six-year samples. SY: six-year samples.

The DEGs of two comparison groups were annotated in the KEGG database, and then an enrichment analysis was conducted. The annotation classification and top 20 enrichment pathways of ASY and SY are shown in Figure 9, respectively. KEGG annotation suggested that most of the DEGs participated in metabolic pathways (51.37%) and the biosynthesis of secondary metabolites (25.23%). Two KEGG pathways (cysteine and methionine metabolism, and carbon fixation in photosynthetic organisms) were the exclusive means between ASY and SY. Moreover, some interesting annotation results revealed that many DEGs participated in starch and sucrose metabolism and the biosynthesis of amino acids, despite their occurrence in the leaves. Interestingly, ABC transporters, also one of the main annotation pathways, may play an important role in transferring these primary components into blubs to be stored for growth in the next year [37,38]. In addition, plant hormone signal transduction combined with the circadian rhythm of plants may synergistically contribute to the phenotypic plasticity of *F. cirrhosa*. Herein, ABC transporters may synergistically regulate the phenotypic plasticity of *F. cirrhosa* by changing the variation in phytohormones owing to the powerful ability of the transporters and annotation results of both ABC transporters and plant hormone signal transduction [39]. A total of 5.67% of DEGs had a close connection with plant–pathogen interaction, aligning with our hypothesis that pathogens may be the main reason for phenotypic plasticity from the reproductive stage to vegetative growth status.

The scatter plots of KEGG enrichment analysis illustrate the top 20 pathways with the most significant *p*-values, in which a high rich factor (a ratio of DEGs annotated in one pathway to the whole genes with annotation information) indicates a deep enrichment degree and a low *p*-value indicated significant enrichment. The comparison group of ASY and SY had two enrichment pathways with extremely low *p*-values but low rich factors, namely the circadian rhythm of the plant and pantothenate and CoA biosynthesis (Figure 9 (below)). Many enrichment pathways were related to amino acid metabolism, which was similar to those observed in the s metabolome results mentioned above.

The annotation classification and top 20 enrichment pathways of ASY and FY are shown in Figure S8A and Figure S8B, respectively. No obvious difference was found in the cellular processes and organismal systems owing to the similar annotation results between the two comparison groups. The metabolic pathways revealed clear annotation differences and similarities. Eleven pathways were identified as common annotation results, including starch and sucrose metabolism, phenylpropanoid biosynthesis, pentose and glucuronate interconversions, oxidative phosphorylation, metabolic pathways, glyoxylate, and dicarboxylate metabolism, glycolysis/gluconeogenesis, galactose metabolism, carbon metabolism, biosynthesis of secondary metabolites, and biosynthesis of amino acids. The comparison group between ASY and FY had unique pathways in pyruvate metabolism, flavonoid biosynthesis, cysteine and methionine metabolism, and carotenoid biosynthesis. The circadian rhythm of plant and flavonoid biosynthesis were the main enriched KEGG pathways based on the annotation of DEGs between ASY and FY. 

**Figure 10:**
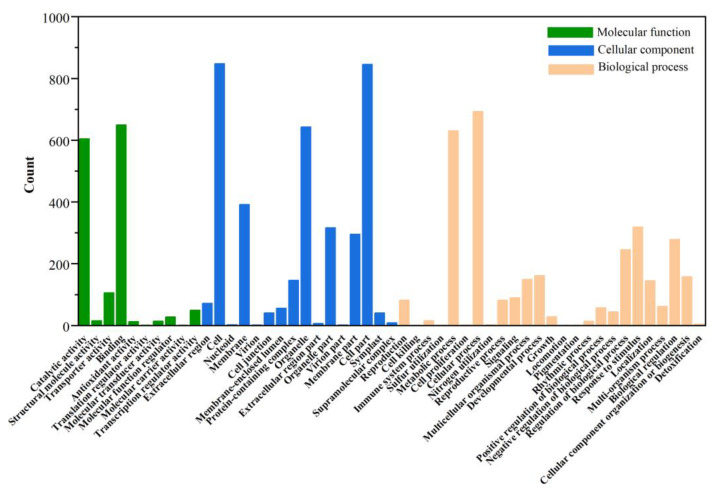
The GO classification of different genes between ASY and SY in *F. cirrhosa*. ASY: aberrant six-year samples. SY: six-year samples.

In addition to KEGG annotation, the GO database contains updated annotation information for DEGs. The annotation results were divided into three categories: cellular components, molecular functions, and biological processes. Detailed annotation information with the number of DEGs and the percentage of DEGs of individual comparison groups are shown in Figure 10. The annotation results were not significantly different between the two groups (ASY and SY and ASY and FY). Further, the DEGs annotation of ASY and FY lacked the functional profiles of translation regulator activity in the functional category of molecular function (Figure S10). Carbon utilization and biological adhesion were missing between ASY and SY.

Owing to the absence of whole genomic data for *Fritillaria*, the molecular mechanism leading to phenotype plasticity was not previously clear. Transcriptome sequencing based on advanced assembly technologies provides vast functional information regarding plant genomes. However, few reports have focused on plant growth instead of the biosynthesis of secondary metabolites, mainly alkaloids [40,41,42,43]. We obtained nearly 20 Gb of raw data from the three phenotypes with different growth ages. The huge genetic profiles combined with public annotation databases, such as KEGG and GO, provide reliable reference information for interpreting the gene function of phenotypic plasticity of *F. cirrhosa* [44]. Those key biosynthesis genes related to flavonoid metabolites were affected by some environmental stress, such as salt stress in *Solanum nigrum*, and the relative genes, such as *SnPAL*, *SnCHS*, and *SnFLS* were upregulated in the stress treatment [45]. Flavonoid biosynthesis was the main enrichment KEGG pathways with low *p*-values (Figure S8) based on the annotation of DEGs between ASY and FY; however, the 22 significantly different genes in flavonoid biosynthesis were upregulated in the ASY phenotype, which may be interpreted as ASY actually suffering from a stress condition. Interestingly, 46 regulated genes participated in starch and sucrose metabolism, including 17 upregulated and 29 downregulated genes. A high expression level (high total FPKM value) was found in FY based on the upregulated DEGs; however, ASY had higher values for the downregulated genes. We hypothesized that the starch and sucrose synthesized in leaves are transferred into the bulbs in a stored form for growth in the following years. Thus, FY has a stronger ability to produce primary metabolites than ASY, which was consistent with the field investigation that the FY bulbs have short cultivation years with stronger vitality. Furthermore, starch and sucrose metabolism contribute to bulblet development and formation in *Sagittaria sagittifolia* [46]. In addition, the bulb samples displayed a tendency for sucrose accumulation at an early stage of bulb development according to the transcriptome analysis of sucrose metabolism in onions of different swelling and development periods [47].

#### 3.3.3. Transcription Factors (TFs) Participate in the Process of Phenotype Plasticity in *F. cirrhosa*

Based on the two TF databases of PInTFDB and PlantTFDB, 35 TFs (TRs) families from 62 DEGs were identified between ASY and SY (Table S9), while 39 TFs (TRs) families encoded by 102 DEGs were found to belong to the comparison group of ASY and FY (Table S10). The gene amounts managing the TF or TR are shown in Figure S10 for the comparison groups among ASY, SY, and FY. Herein, CSD, GNAT, MADS-M-type, and NF-YB are unique differential TFs (TRs) belonging to the ASY and SY comparison groups. The BSD was encoded by upregulated genes while DDT was encoded by one downregulated gene. Further, IWS1 was encoded by one downregulated gene, and TAZ was encoded by one downregulated gene, which were exclusive TFs (TRs) between the ASY and FY samples. The comparison groups of the SY and FY samples had proprietary TFs (TRs) that comprised HB-HD-ZIP encoded by one upregulated and one downregulated gene, PLATZ encoded by upregulated genes, and SBP encoded by two upregulated genes.

Many TFs and TRs have been observed among the three phenotypes of *F. cirrhosa*, and critical roles in phenotypic plasticity. In fact, these TFs and TRs have been demonstrated to be involved in plant germination, sprouting, and even plant dormancy [48]. These factors also play important roles in controlling and regulating various biological processes and metabolites. Phenotypic plasticity might be directly related to some vital TFs or TRs, such as MADS-box, MYB, bHLH, WRKY, NAC, C2C2-GATA, and GRAS. MADS-box genes have been validated for the potential ability to regulate growth–dormancy cycles and flowering in perennials [49]. Two MADS-box TFs (MADS-MIKC and MADS-M-type) were found to participate in phenotype plasticity, which emerged between ASY and SY, and between SY and FY. There were no MADS-box TFs between ASY and FY, two vegetative growth statuses of *F. cirrhosa* without aerial stems and flowers. Other similar TFs, such as Trihelix, AR1, HB-HD-ZIP, and HRT, showed the same validation evidence, as these TFs may play an important role in the regenerative stage of *F. cirrhosa*. Four TFs/TRs (BSD, DDT, IWS1, and TAZ) were expressed in the comparison group of ASY and FY; these TFs/TRs may play a vital role in the vegetative period. The MYB and bHLH TFs families are widely found in plants and are involved in many functions, including basal plant development and metabolism, flower organ development, and plant hormone response [50]. Based on the annotation results, MYB exists between ASY and SY, ASY and FY, which cannot explain the role of MYB role in perennial growth from the vegetative stage to the regenerative status owing to the differences in growing years. Similarly, 18 unigenes were annotated as bHLH among the two comparison groups (ASY, SY, and FY), and the upregulated and downregulated unigenes were found to be similar. WRKY is a crucial TF family in plant defenses against various pathogenic infections [51]. Two DEGs were upregulated between ASY and FY. Further, seven DEGs encoding the TF were downregulated, which indicated that the SY phenotype may possess stronger resistance than ASY. Thus, ASY may be the phenotype of SY that suffers from pathogens, leading to the growth of small bulbs, which cannot germinate into flower buds in the next generation [52].

Based on the results of the present comparison and our field investigation, biotic stress might be the leading cause of the phenotypic plasticity of *F. cirrhosa*, which aligned with our hypothesis. However, this conclusion must be further examined. In addition, an investigation on endophytes and rhizospheric microorganisms must be performed. Whether phenotypic plasticity affects bioactive components, and the role of plant hormones has been previously assessed owning the enrichment pathways involved in the biosynthesis of secondary metabolites and plant hormone signal transduction.

## 4. Conclusions

In the current study, we analyzed and sequenced the metabolome and transcriptome of two phenotypes of *F. cirrhosa* from the same cultivation years. Metabolome analysis indicated that amino acids and derivatives, and free fatty acids may be the main chemical categories resulting in phenotypic plasticity, where the phenotype varies from the vegetative to the productive stage. Transcriptome analysis revealed that the DEGs among the three phenotypes mainly participated in amino acid biosynthesis. Moreover, two annotation pathways in starch and sucrose metabolism and plant–pathogen interactions were identified. Overall, this study provides new insights into the phenotypic variation mechanisms of *F. cirrhosa*.; such variation may be due to the regulation of the synthesis of starch, amino acids, and other substances in bulbs caused by resistance to pathogens and stressful environments. However, further exploration and verification must be carried out in future research.

## Figures and Tables

**Table 1:** The detailed amount of difference metabolites among different comparison phenotypes of *F. cirrhosa* (ASY is control phenotype for calculating the up- and downregulated components).

Metabolite Category	ASY and SY		ASY and FY	
	Up	Down	Up	Down
Phenolic acids	21	28	40	7
Amino acids and derivatives	4	36	11	39
Lipids	1	36	7	3
Organic acids	6	11	9	13
Nucleotides and derivatives	2	11	11	11
Others	12	4	1	5

## Data Availability

We have uploaded the RNA sequencing data to NCBI, and the link is https://www.ncbi.nlm.nih.gov/bioproject/PRJNA849777 (accessed on 6 November 2022). The data sets supporting the results of this article are include within the article and supplementary table.

## Supplementary Materials

The following supporting information can be downloaded at: https://www.mdpi.com/article/10.3390/cells11233844/s1, Figure S1: The results of OPLS-DA using metabolic data between ASY and FY phenotypes of *F. cirrhosa*. (A) Score plot of OPLS-DA; (B) S-plot; (C) The results of 200 times permutation test; Figure S2: The selection of different metabolites and comparison of amino acids and derivatives between ASY and FY phenotypes of *F. cirrhosa*. (A) The volcano plot; (B) Chord diagram of differential metabolites; (C) Heatmaps of metabolites; Figure S3: The KEGG classification plot of difference metabolites between ASY and SY in *F. cirrhosa*; Figure S4: The variation of different metabolites in the biosynthesis of amino acids between ASY and SY in *F. cirrhosa*; Figure S5: The KEGG classification plot of difference metabolites between ASY and FY in *F. cirrhosa*; Figure S6: The main annotation results of different metabolites between ASY and FY in *F. cirrhosa*. (A) KEGG classification analysis. (B) The enrichment analysis; Figure S7: Venn plot of different genes among three comparison groups of *F. cirrhosa*; Figure S8: The main annotation results of different genes between ASY and FY in *F. cirrhosa*; (A) KEGG classification analysis. (B) The enrichment analysis; Figure S9: The GO classification of different genes between ASY and FY in *F. cirrhosa*; Figure S10: Transcription factor and transcriptional regulator of two comparison groups including ASY and SY (A) and ASY and FY (B). Table S1: The detailed metabolites information detected in the bulbs of *F. cirrhosa*; Table S2: The difference metabolites information detected in the bulbs of *F. cirrhosa* between ASY and SY; Table S3: The difference metabolites information detected in the bulbs of *F. cirrhosa* between ASY and FY; Table S4: Results of RNA quality assessment of nine leaves samples of *F. cirrhosa*; Table S5: RNA sequencing results of nine leave samples of *F. cirrhosa*; Table S6: All significant difference genes between ASY and SY of *F. cirrhosa*; Table S7: All significant difference genes between ASY and FY of *F. cirrhosa*; Table S8: Nr annotation results similar to the same genus of *Fritillaria*; Table S9: Transcription factors of differentially expression between ASY and SY of *F. cirrhosa*; Table S10: Transcription factors of differentially expression between ASY and FY of *F. cirrhosa*.

## Abbreviation

ASY	Aberrant six-year samples
DEGs	Differentially expressed genes
FDR	False discovery rate
FPKM	Kilobase of transcript per million fragments mapped
FY	Four-year samples
GO	Gene Ontology
HCA	Hierarchical cluster analysis
KEGG	Kyoto Encyclopedia of Genes and Genomes
Nr	NCBI non-redundant protein sequences
OPLS-DA	Orthogonal partial least squares-discriminant analysis
PCA	Principal component analysis
SY	Six-year samples
TFs	Transcription factors
UPLC–ESI–MS/MS	Ultra-performance liquid chromatography-electrospray ionization-tandem mass spectrometry
VIP	Variable importance in projection
