# Supplementary material for: Primary Investigation of Phenotypic Plasticity in Fritillaria cirrhosa Based on Metabolome and Transcriptome Analyses
Source: Cells. 2022 Nov 30;11(23):3844. doi: 10.3390/cells11233844 (PMC9736200; doi:10.3390/cells11233844)
Supplement: Supplementary file 1 [file cells-11-03844-s001.zip › Supplementary Material.pdf]

Supplementary Material

Primary Investigation of Phenotypic Plasticity in *Fritillaria cirrhosa* Based on Metabolome and Transcriptome Analyses

Ye Wang [1], Huigan Xie [2], Tiechui Yang [2], Dan Gao [1,*] and Xiwen Li [1,*]

[1] Institute of Chinese Materia Medica, China Academy of Chinese Medical Sciences, Beijing 100700, China
[2] Nin Jiom Medicine Manufactory (Hong Kong) Limited, Hong Kong 999077, China
* Correspondence: dgao@icmm.ac.cn (D.G.); xwli@icmm.ac.cn (X.L.); Tel.: +86-10-8408-4107

1 Supplementary Data

Supplementary data were uploaded separately on submission in the form of .xlsx.

2 Supplementary Figures

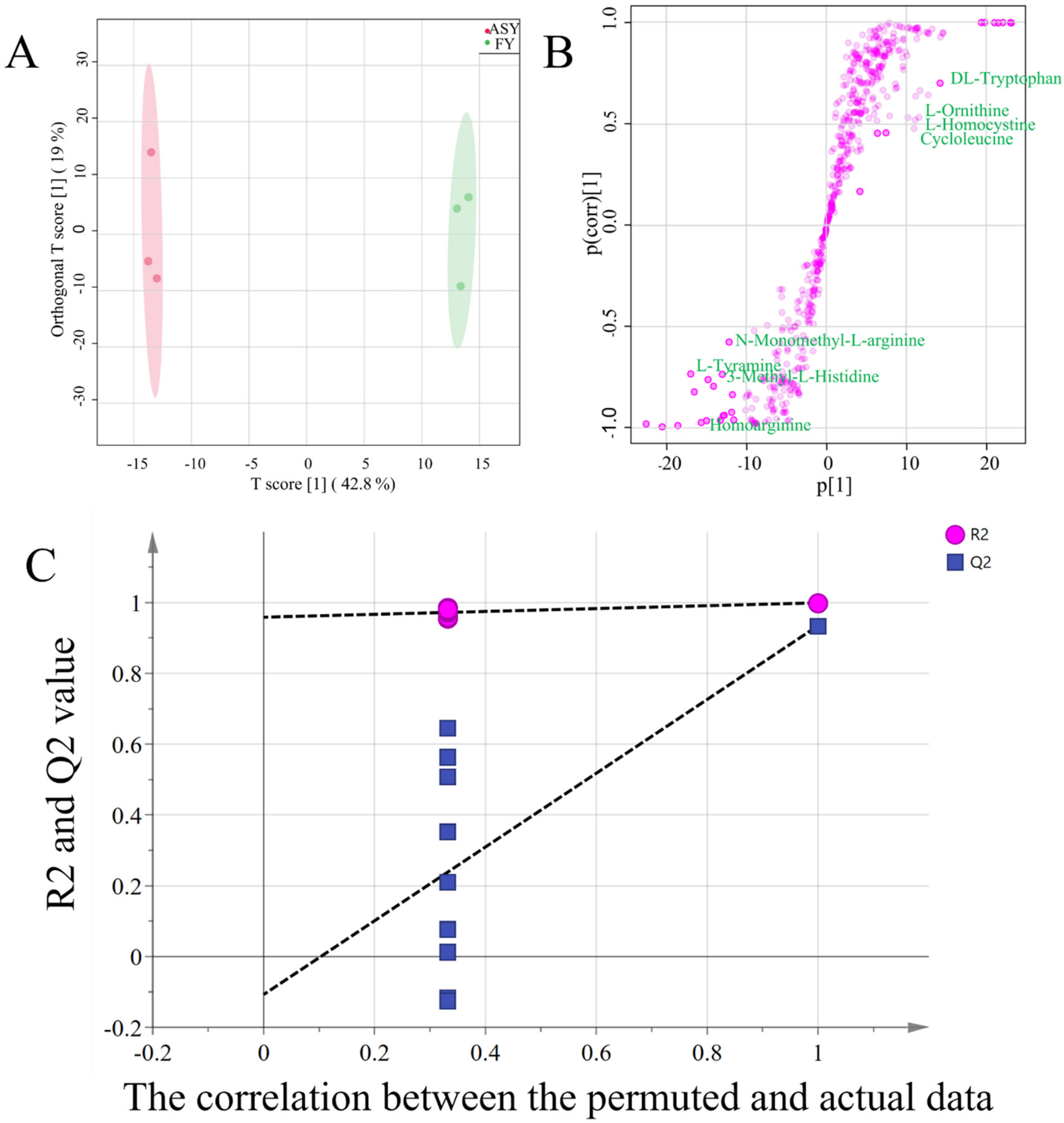

Figure S1. The results of OPLS-DA using metabolic data between ASY and FY phenotypes of *F. cirrhosa*. (A) Score plot of OPLS-DA; (B) S-plot; (C) The results of 200 times permutation test. ASY: Aberrant six-year sample. FY: Four-year samples.

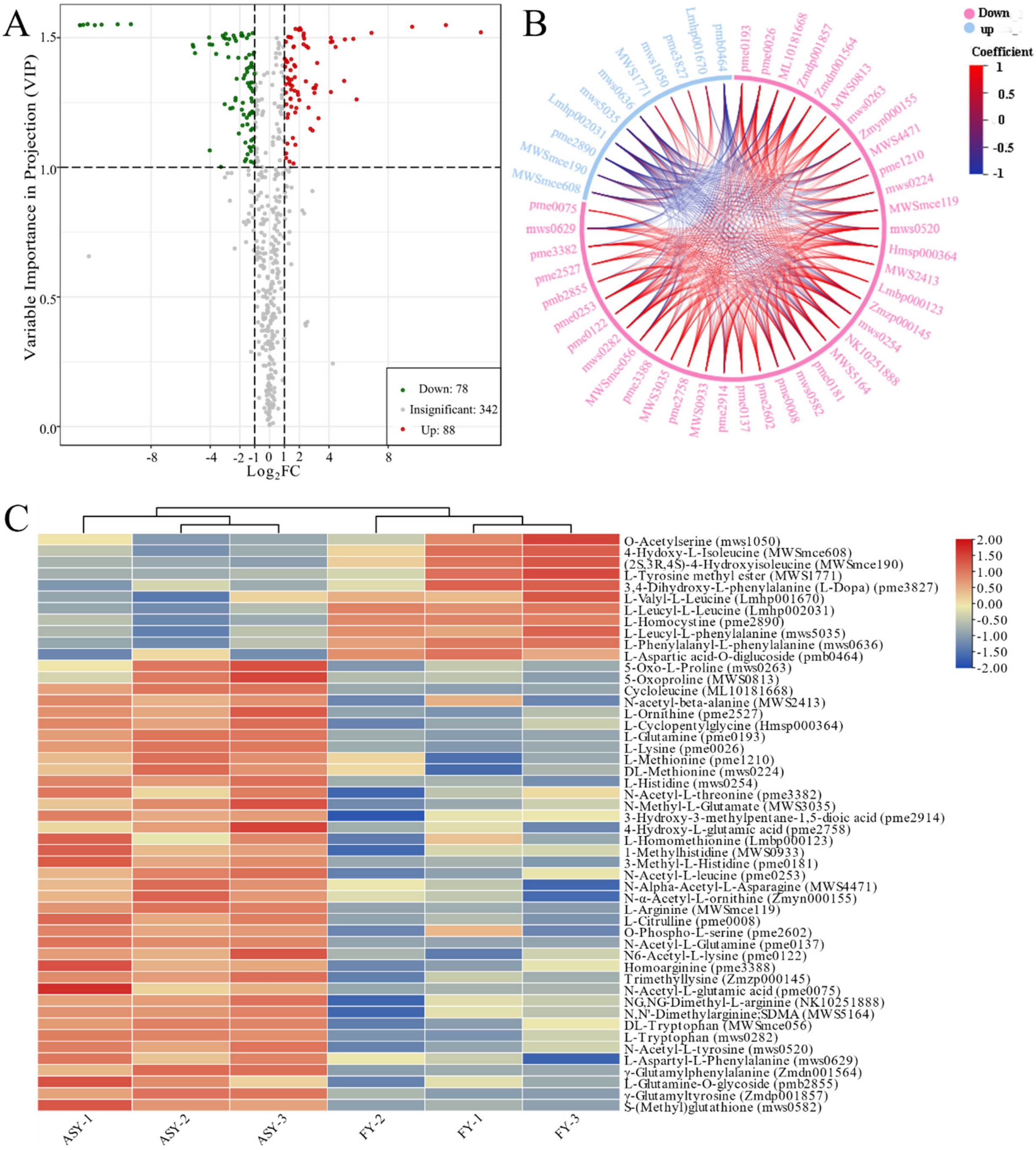

Figure S2. The selection of different metabolites and comparison of amino acids and derivatives between ASY and FY phenotypes of *F. cirrhosa*. (A) The volcano plot; (B) Chord diagram of differential metabolites, which full names are the same as those of heatmap; (C) Heatmap of amino acids metabolites. ASY: Aberrant six-year samples. FY: Four-year samples.

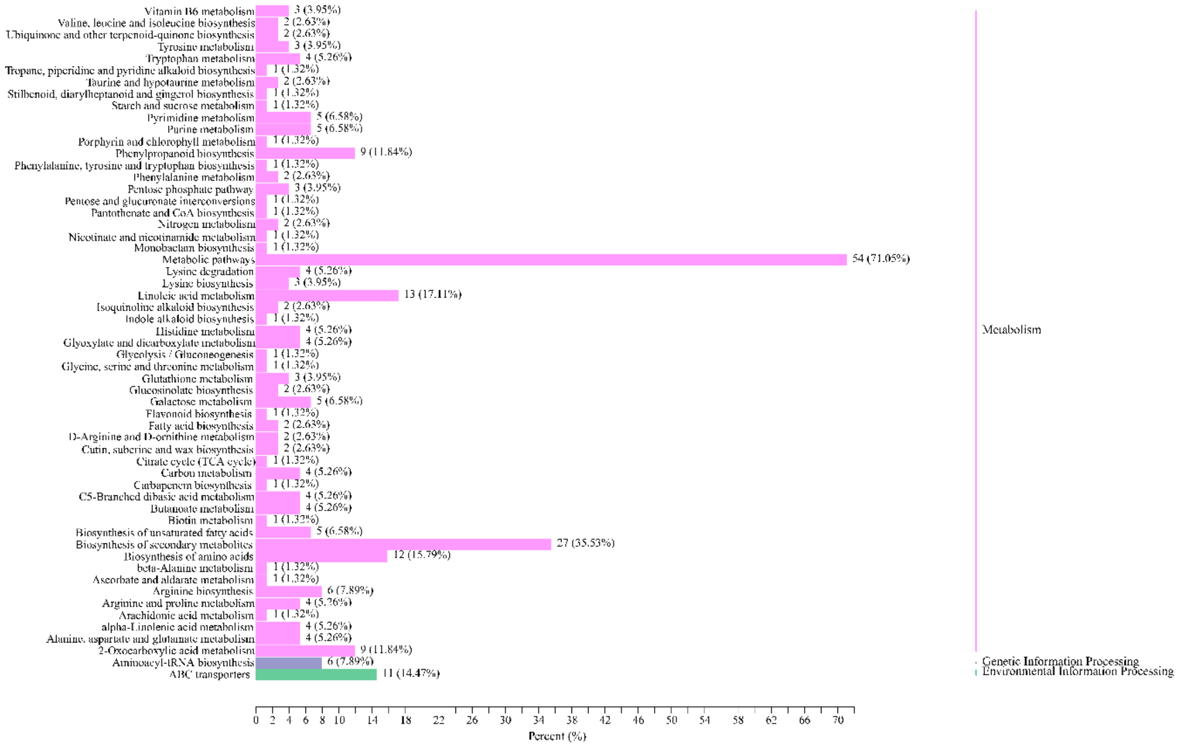

Figure S3. The KEGG classification plot of difference metabolites between ASY and SY in *F. cirrhosa*. ASY: Aberrant six-year samples. SY: Six-year samples.

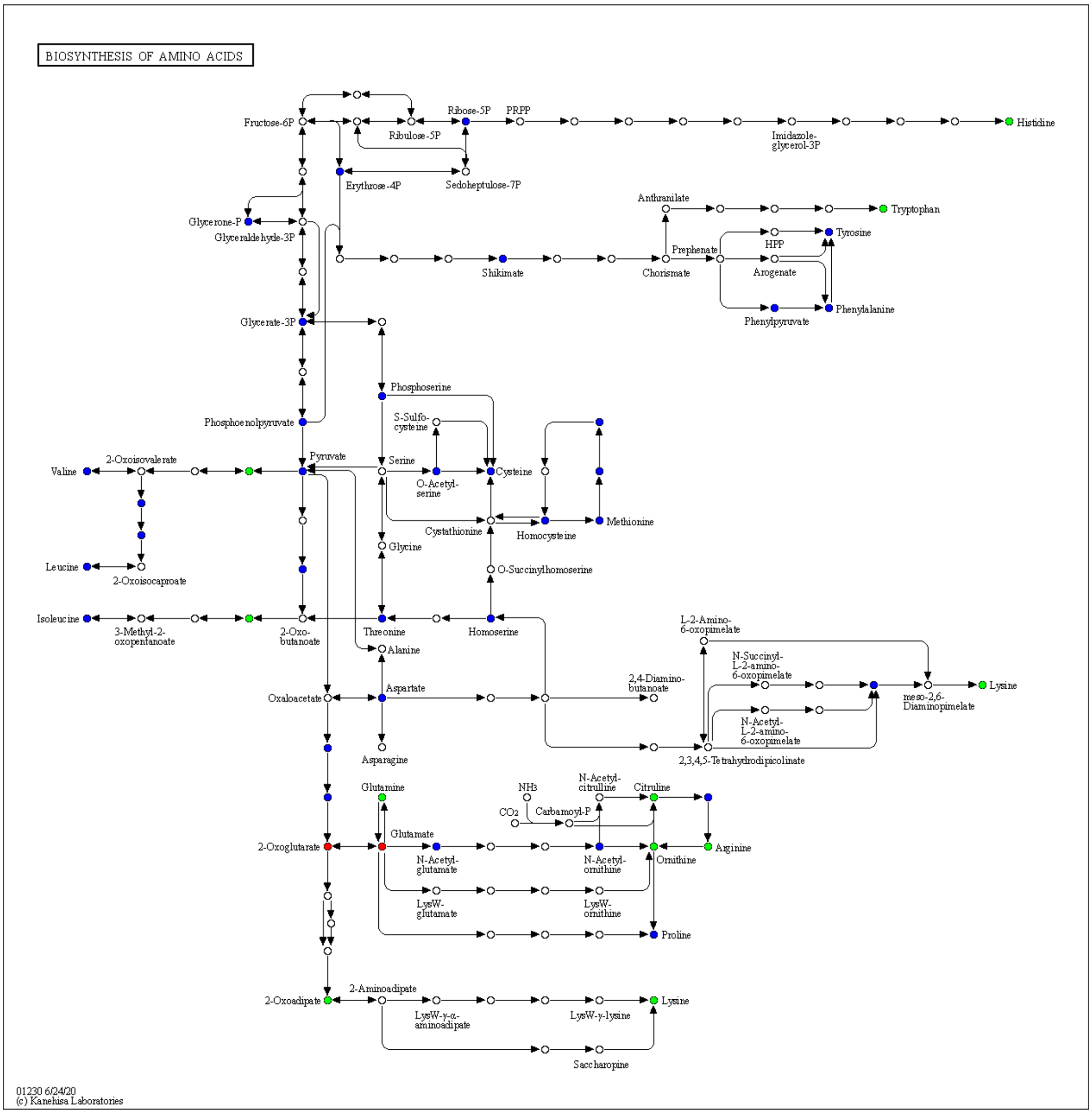

Figure S4. The variation of difference metabolites in the biosynthesis of amino acids between ASY and SY in *F. cirrhosa*. ASY: Aberrant six-year samples. SY: Six-year samples. Red point means the significantly up-regulated metabolites, green point means the significantly down-regulated metabolites, blue point means these metabolites have significantly difference.

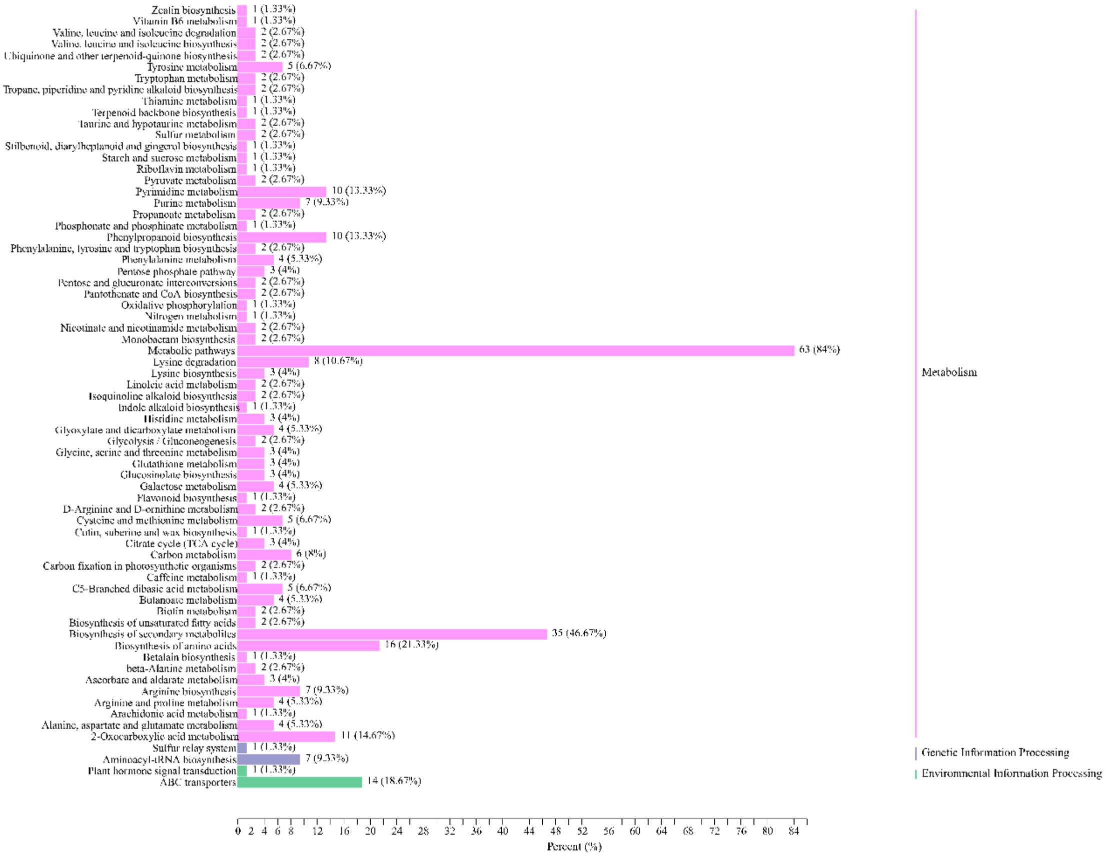

Figure S5. The KEGG classification plot of difference metabolites between ASY and FY in *F. cirrhosa*. ASY: Aberrant six-year samples. FY: Four-year samples.

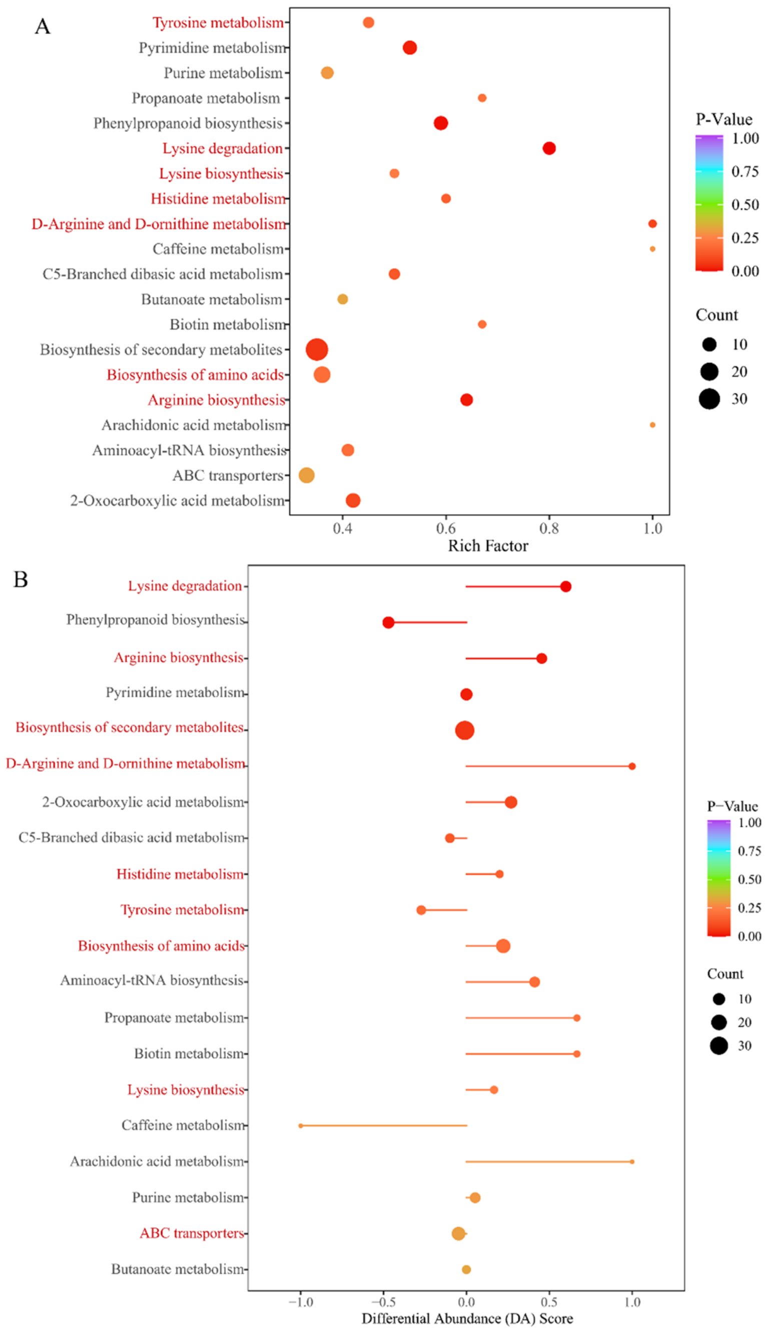

Figure S6. The main annotation results of difference metabolites between ASY and FY in *F. cirrhosa*. (A) KEGG classification analysis. (B) The enrichment analysis. ASY: Aberrant six-year samples. FY: Four-year samples.

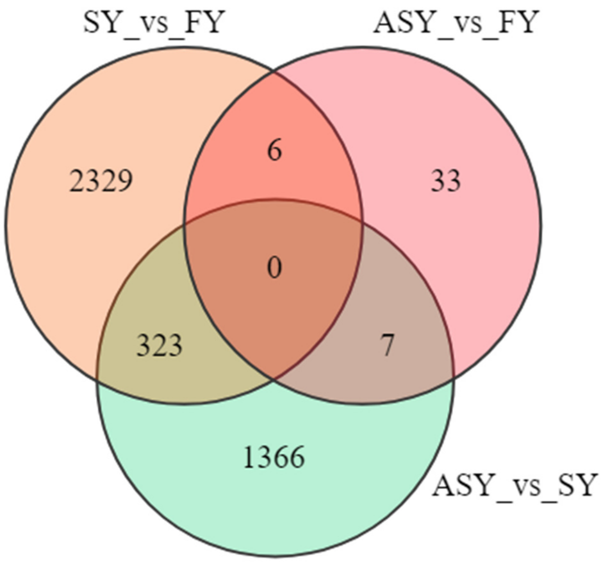

Figure S7. Venn plot of difference genes among three comparison groups of *F. cirrhosa*. FY: Four-year samples. SY: Six-year samples. ASY: Aberrant six-year samples.

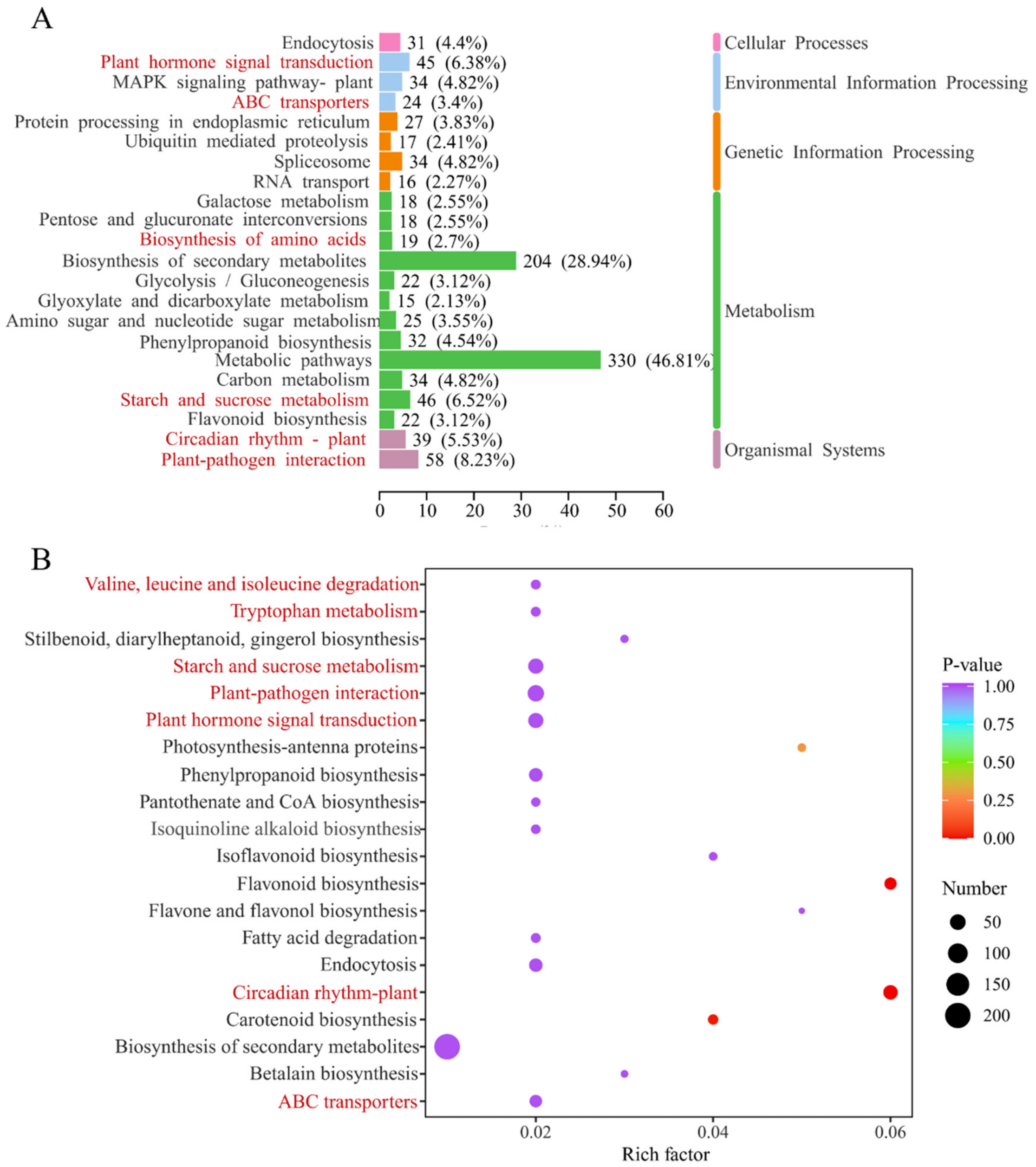

Figure S8. The main annotation results of difference genes between ASY and FY in *F. cirrhosa*. (A) KEGG classification analysis. (B) The enrichment analysis. ASY: Aberrant six-year samples. FY: Four-year samples.

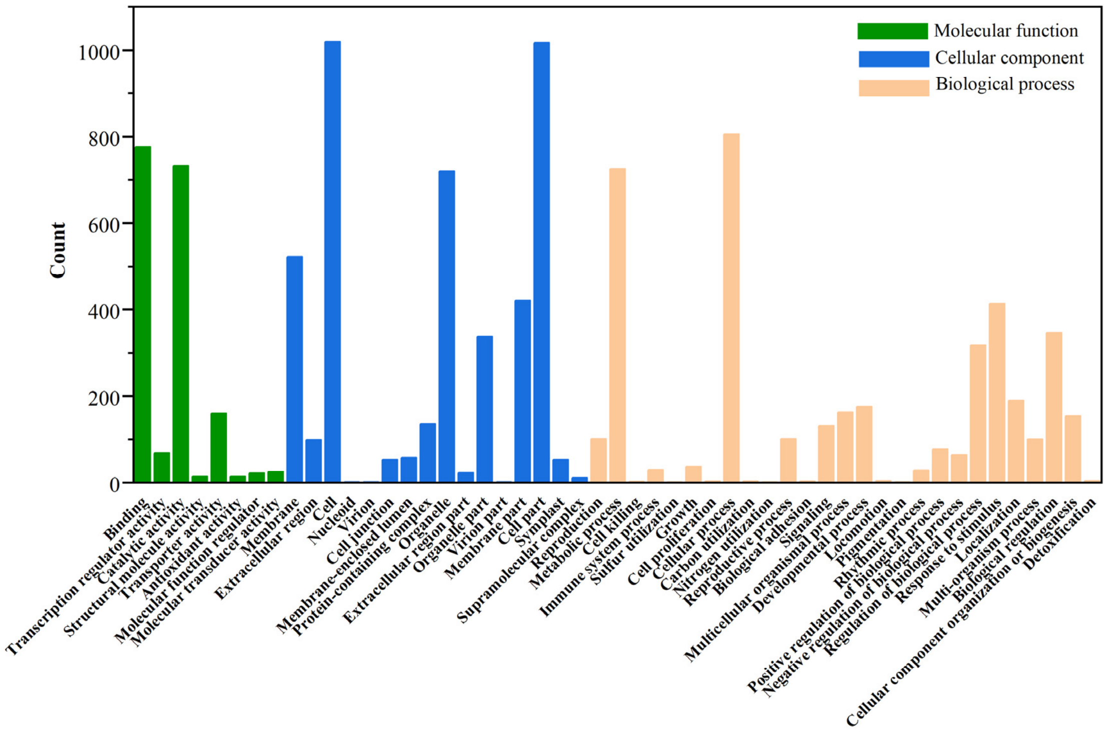

Figure S9. The GO classification of difference genes between ASY and FY in *F. cirrhosa*. ASY: Aberrant six-year samples. FY: Four-year samples.

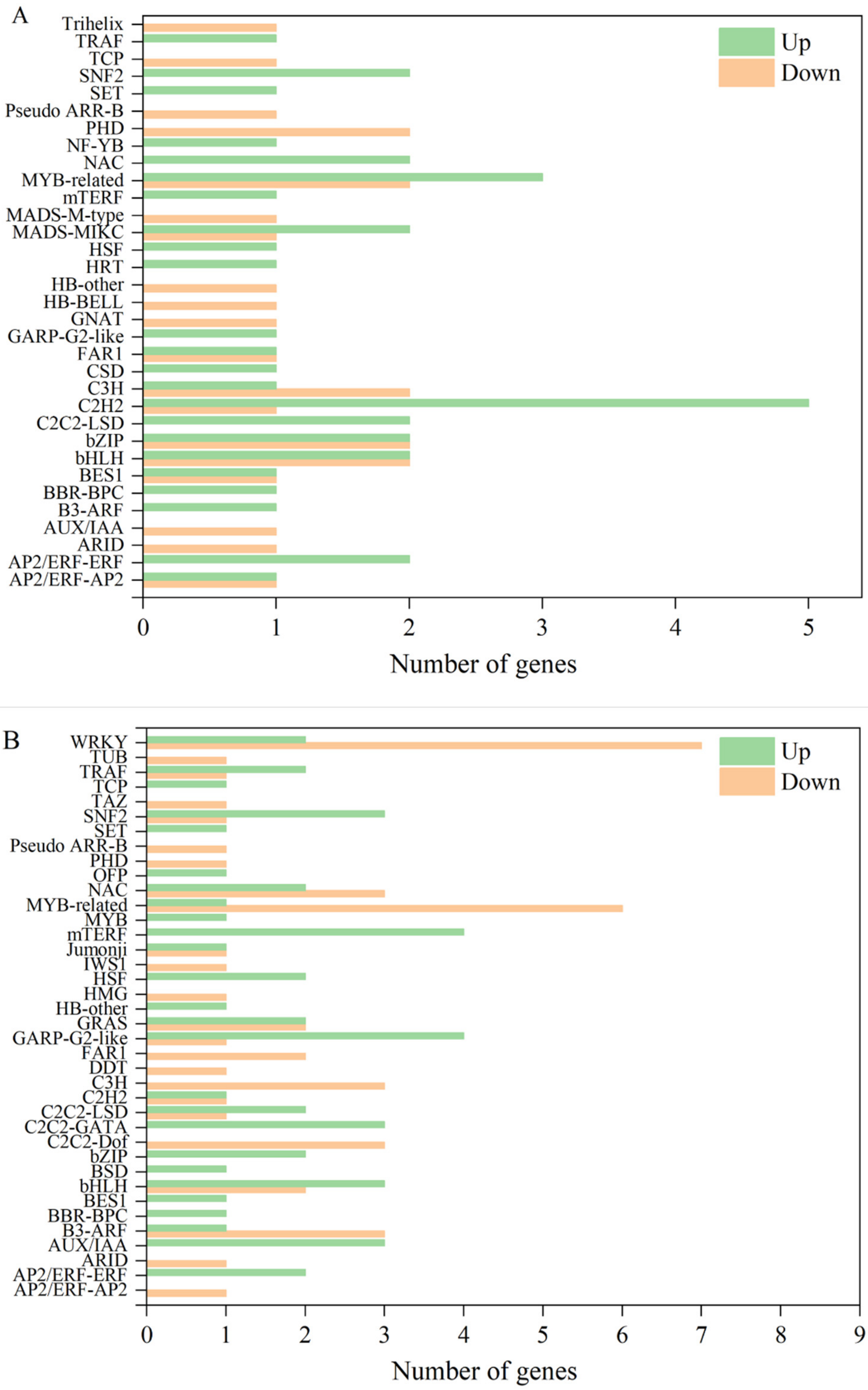

Figure S10. Transcription factor and transcriptional regulator of two comparison group including ASY&SY (A) and ASY&FY (B). FY: Four-year samples. SY: Six-year samples. ASY: aberrant six-year samples.
